# Soybean Roots and Soil From High- and Low-Yielding Field Sites Have Different Microbiome Composition

**DOI:** 10.3389/fmicb.2021.675352

**Published:** 2021-11-30

**Authors:** Ananda Y. Bandara, Dilooshi K. Weerasooriya, Ryan V. Trexler, Terrence H. Bell, Paul D. Esker

**Affiliations:** ^1^Department of Plant Pathology & Environmental Microbiology, The Pennsylvania State University, University Park, PA, United States; ^2^Intercollege Graduate Degree Program in Ecology, The Pennsylvania State University, University Park, PA, United States

**Keywords:** soybean, spatial yield variation, microbiome, metagenomics, fungal ITS, bacterial 16S rRNA, metabarcoding

## Abstract

The occurrence of high- (H) and low- (L) yielding field sites within a farm is a commonly observed phenomenon in soybean cultivation. Site topography, soil physical and chemical attributes, and soil/root-associated microbial composition can contribute to this phenomenon. In order to better understand the microbial dynamics associated with each site type (H/L), we collected bulk soil (BS), rhizosphere soil (RS), and soybean root (R) samples from historically high and low yield sites across eight Pennsylvania farms at V1 (first trifoliate) and R8 (maturity) soybean growth stages (SGS). We extracted DNA extracted from collected samples and performed high-throughput sequencing of PCR amplicons from both the fungal ITS and prokaryotic 16S rRNA gene regions. Sequences were then grouped into amplicon sequence variants (ASVs) and subjected to network analysis. Based on both ITS and 16S rRNA gene data, a greater network size and edges were observed for all sample types from H-sites compared to L-sites at both SGS. Network analysis suggested that the number of potential microbial interactions/associations were greater in samples from H-sites compared to L-sites. Diversity analyses indicated that site-type was not a main driver of alpha and beta diversity in soybean-associated microbial communities. L-sites contained a greater percentage of fungal phytopathogens (ex: *Fusarium*, *Macrophomina*, *Septoria*), while H-sites contained a greater percentage of mycoparasitic (ex: *Trichoderma*) and entomopathogenic (ex: *Metarhizium*) fungal genera. Furthermore, roots from H-sites possessed a greater percentage of *Bradyrhizobium* and genera known to contain plant growth promoting bacteria (ex: *Flavobacterium*, *Duganella*). Overall, our results revealed that there were differences in microbial composition in soil and roots from H- and L-sites across a variety of soybean farms. Based on our findings, we hypothesize that differences in microbial composition could have a causative relationship with observed within-farm variability in soybean yield.

## Introduction

Spatial heterogeneity in soil characteristics within agricultural fields is a frequently observed phenomenon that can influence yield and other crop traits (Khakural et al., 1996; Jaynes and Colvin, 1997; Paz et al., 1998; Kravchenko and Bullock, 2000; Vollmann et al., 2000; Batchelor et al., 2002; Shahandeh et al., 2005). Spatial field heterogeneity typically implies the occurrence of soil fertility gradients, owing to trends in different soil parameters, such as soil physical properties (Becher, 1995), element concentration (Berndtsson and Bahri, 1995), organic carbon content (Ball et al., 1993), and water and nitrogen content (Mulla et al., 1992; Wade et al., 1996). Spatial field heterogeneity can be closely linked with spatial variability in crop yields. In fact, understanding observed yield variability within corn and soybean fields is one of the most captivating problems in production research (Batchelor et al., 2002).

The site-scale spatial heterogeneity of various soil characteristics and their relationships with soybean yields have been previously reported in several locations including Iowa (Kaspar et al., 2004; Rogovska et al., 2007), Mississippi (Cox et al., 2003), and Michigan (Jiang and Thelen, 2004). These studies indicated that site-scale heterogeneity in soil factors, such as pH, P, K, slope of the site, site elevation, and soil texture explained the observed variability in soybean yield. Mann et al. (2019) studied bulk soil samples from farmer-designated “good” vs. “poor” sites across 34 farms in Maritime Canada and used Cornell’s Comprehensive Assessment of Soil Health (CASH), which integrates soil texture, available water capacity, surface and subsurface hardness, wet aggregate stability, autoclaved-citrate extractable soil protein, and soil respiration (Moebius-Clune et al., 2016), combined with phospholipid fatty acid analysis (PLFA), and conventional soil chemical analysis to assess the collected samples. Although CASH and PLFA assays did not statistically differentiate farmer-designated “good” and “poor” sites, differences between good and poor sites were evident when focusing on certain soil nutrients, pH, and organic matter. For example, the authors found that farmer-identified good sites contained significantly greater levels of B, Ca, Mg, and higher pH, compared to poor sites.

In order to understand the causes behind the occurrence of high- and low-yielding field sites (=within-farm-spatial-variation of soybean yields) across, we conducted a soil survey by using 14 farms within the Pennsylvania soybean on-farm network (Bandara et al., 2020b). Bulk soil samples were collected and a number of soil chemical, physical, topological, and biological factors were assessed. Samples from two site types did not exhibit significant differences based on the assessed factors. Our findings indicated that the causes behind within-farm-spatial-variation of soybean yields across selected farms are complex and investigations using additional variables such as soil and plant-associated microbial composition may be useful in understanding within-site variability in yield.

Although the potential association of fungal and prokaryotic composition with within-site spatial variability in crop yield has not been previously demonstrated, differences in fungal and prokaryotic composition have been reported as a function of space-related variables. For example, Srour et al. (2017) found significant differences in prokaryotic and fungal composition between soils collected from healthy areas and areas where sudden death syndrome symptoms were visible. Kasel et al. (2008) observed that sites differing in land use within the same location also differed in soil fungal composition. Furthermore, substantial differences in microbial composition have been observed in relation to different agronomic/cultural practices, such as tillage (Yin et al., 2010; Dorr de Quadros et al., 2012; Mathew et al., 2012), crop rotation (Yin et al., 2010; Dorr de Quadros et al., 2012; Mathew et al., 2012), pesticide/herbicide use (Crouzet et al., 2010; Lo, 2010; Banks et al., 2014), and organic/conventional agriculture (Moeskops et al., 2010; Sugiyama et al., 2010; Schmid et al., 2011).

The composition of microorganisms in the rhizosphere, which is the region of the soil in contact with plant roots, can affect plant performance. If host plants can capitalize on the microbial services presented by the microbiome, agricultural productivity could be augmented through more fully harnessing beneficial functions. Microbial services may include the production of phytohormones (Ping and Boland, 2004), improved tolerance to abiotic and biotic stresses (Redman et al., 2002), induction of the plant innate immune response mechanisms (Jain et al., 2011), delivery of nutrients (Janos, 2007), modification of plant functional traits (Friesen et al., 2011), or tissue chemistry (Larsen et al., 2006). All of these services are directly related to microbial composition. Therefore, spatial differences in microbial structure could potentially contribute to differential plant performance, manifested as yield variation either at a narrow geographic scale (e.g., site-to-site variation within the farm) or at a broader geographic scale (e.g., farm-to-farm variation). In this study, our objective was to investigate the association between site-to-site spatial variation of soybean yield within a farm (hereafter referred to as within-farm-spatial-variation of soybean yields) and fungal/prokaryotic composition in bulk soil, rhizosphere soil, and soybean roots using an amplicon-sequencing approach.

## Materials and Methods

### Experimental Locations, Sample Collection, and Sample Processing

**FIGURE 1:**
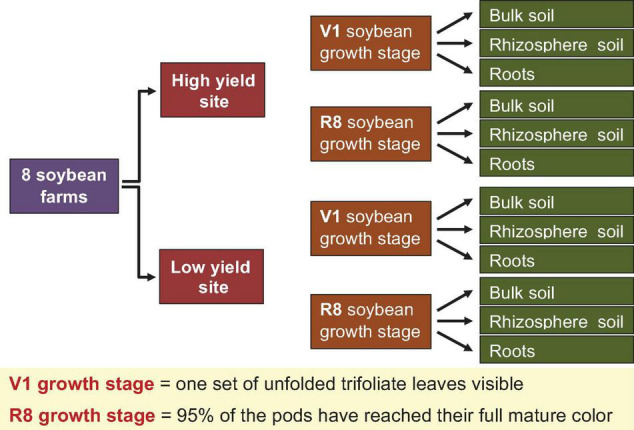
Strategy adopted for sample collection. Bulk soil, rhizosphere soil, and roots were collected from one farmer designated high and one low yield site at V1 (one set of unfolded trifoliate leaf is visible) and R8 (95% of the pods have reached their mature color) soybean growth stages from eight soybean farms in Pennsylvania.

Eight farms located in eight Pennsylvania counties (Cambria, Bedford, Bucks, Butler, Lancaster, Mercer, Northumberland, Tioga) within the “Pennsylvania Soybean On Farm Network” were randomly chosen for this study. Figure 1 summarizes the sampling strategy that was used for sample collection. At each farm, farmer-designated high- and low-yield sites were defined and the Global Positioning System (GPS) coordinates were recorded, as well as the sampling date (Supplementary Table 1). Within each site type, five soybean plants were randomly selected and surface debris around the plant was removed. The aboveground plant organs were aseptically removed. Using a shovel, plants were removed with the root ball intact. The size of each root ball was ∼15–20 cm^3^. Entire root balls were transported to the Esker Lab at The Pennsylvania State University on dry ice on the same day that they were sampled. Sampling was performed at two soybean growth stages (SGS) (V1 = one set of unfolded trifoliate leaf is visible, and R8 = 95% of the pods have reached their mature color). These two growth stages were selected to represent early vegetative and late reproductive stages of the soybean lifecycle.

Each root ball was fractionated into bulk soil, rhizosphere soil, and roots. For bulk soil, loose soil was manually removed from the root ball by kneading and shaking the soil with sterile gloves on to a sterile work surface. Accumulated soil was homogenized manually and approximately 20 g was placed into a 50 ml sterile Falcon tube. Subsequently, the left-over soil around the root system was removed by patting roots with a sterile spatula to reach rhizosphere soil. Soil aggregates that extended up to 1 mm from the root surface were carefully collected into a 50 ml Falcon tube (approximately 5–10 g) and these were designated as rhizosphere soil. From the remaining root system, secondary roots were excised using a sterile scalpel and placed in a sterile 50 ml Falcon tube containing ice cold 25 ml phosphate buffered saline (doi: 10.1101/pdb.rec8247 Cold Spring Harb. Protoc. 2006). Only secondary roots were collected across all root balls for consistency. Tubes were vortexed at maximum speed for 20 s, which released most of the leftover soil particles from the roots and turned the buffer turbid. The turbid buffer was then decanted and filled with fresh, ice cold PBS (25 ml) and vortexed as indicated above. The buffer was decanted, and this process was repeated five times, although by the end of the fourth cycle, the PBS solution was typically clear, meaning that there were no visible soil particles in the wash buffer. After the last cycle, the remaining buffer was removed from root sample using Kimwipes. Roots were then placed in a new sterile 50 ml Falcon tube. Tubes containing bulk soil, rhizosphere soil, and roots were stored at −80°C.

### DNA Extraction, PCR Amplification, and High-Throughput Sequencing

All samples were lyophilized for 48 h prior to DNA extraction. Lyophilization is particularly important for efficient DNA extraction from root samples. However, for the sake of consistency, soil samples were also subjected to lyophilization. DNA was extracted from 0.25 to 0.30 g of bulk soil, rhizosphere soil, and roots using the Macherey-Nagel NucleoSpin^®^ 96 Soil DNA Isolation Kit (Macherey-Nagel GmbH & Co. KG, Düren, Germany).

Initial PCR reactions were performed targeting the ITS1 region of the ribosomal gene using universal primers ITS1F (5′-CTTGGTCATTTAGAGGAAGTAA-3′) and 58A2R (5′-CTGC GTTCTTCATCGAT-3′) (Gardes and Bruns, 1993; Martin and Rygiewicz, 2005). As described by Bell et al. (2016), both primers were modified to include overhangs needed for index attachment (ITS1F-Illu = TCGTCGGCAGCGTCAGATGTGTATAAGAGACAGCT TGGTCAT TTAGAGGAAGTAA; 58A2R-Illu = GTCTCGTGG GCTCGGAGATGTGTATAAGAGACAGCTGCGTTCTTCATC GAT). Initial 16S rRNA gene PCR reactions were performed according to the 16S Metagenomic Sequencing Library Preparation guide (part no. 15044223 rev. B) with some modifications (Howard et al., 2017). The universal prokaryotic primers 515F (5′-GTGYCAGCMGCCGCGGTAA-3′) and 806R (5′-GGACTACNVGGGTWTCTAAT-3′) were used for 16S rRNA gene amplifications (Herlemann et al., 2011). Similar to the ITS primers, both 16S primers were also modified to include overhangs needed for index attachment (515F-Illu = TCGTCGGCAGCGTCAGATGTGTATAAGAGACAGGT GYCAGCMGCCGCGGTAA; 806R-Illu = GTCTCGTGGGCT CGGAGATGTGTATAAGAGACAGGGACTACNVGGGTWTC TAAT).

PCR reactions occurred in 30 μl volumes comprised of 0.3 μl (=1.5 U) of HotMaster Taq DNA Polymerase (QuantaBio, Beverly, MA), 3 μl of 10x Taq Buffer with 25 mM Mg^2+^ (QuantaBio, Beverly, MA), 1.5 μl of each primer at 10 μM, 0.6 μl of 10 mM dNTP mix (New England Biolabs Inc., Ipswich, MA), 20.6 μl of H_2_O, and 2.5 μl of template DNA. PCR cycling for fungal ITS amplicons was performed using the following protocol: 94°C for 3 min; 35 cycles of 94°C for 20 s, 45°C for 30 s and 72°C for 45 s; with a final elongation at 72°C for 5 min. For 16S rRNA gene amplifications, the reaction mix was the same as described above. PCR cycling for 16S rRNA gene amplicons was performed using the following protocol: 94°C for 2 min; 25 cycles of 94°C for 20 s, 55°C for 20 s, and 72°C for 30 s; and 72°C for 5 min for the final elongation.

PCR products were run on E-Gel™ 96 Gels with SYBR™ Safe DNA Gel Stain (Thermo Fisher Scientific, Waltham, MA) with E-Gel™ Low Range Quantitative DNA Ladder (Thermo Fisher Scientific, Waltham, MA) to visualize/confirm the PCR products/bands (fungal ITS1 ∼ 380 bp, prokaryotic 16S rRNA ∼ 420 bp).

The initial amplicon cleanup was carried out in clear 96-well plates using the Mag-Bind^®^ TotalPure NGS Kit (Omega Bio-Tek Inc., Norcross, GA). Cleaned PCR amplicons were then subjected to a second round of PCR to attach Illumina Nextera-compatible barcode and adaptors to the amplicons. PCR reactions occurred in 25 μl volumes in 96-well plates and were comprised of 5 μl of PCR product (from the first round of PCR), 2.5 μl of forward and reverse primers (i5 and i7, each at 10 μM) with designated barcodes for overhang attachment, 0.3125 μl (=1.5625 U) of HotMaster Taq DNA Polymerase (QuantaBio, Beverly, MA), 3.125 μl of 10x Taq Buffer with 25 mM Mg^2+^ (QuantaBio, Beverly, MA), 0.625 μl of 10 mM dNTP mix (New England Biolabs Inc., Ipswich, MA), and 10.9375 μl of water. Conditions for index attachment PCR cycling were as follows: 98°C for 1 min; 8 cycles of 98°C for 15 s, 55°C for 30 s, and 72°C for 20 s; and 72°C for 5 min for final elongation.

All barcoded amplicons were normalized (∼25 ng) using the SequalPrep Normalization Kit (Thermo Fisher Scientific, Waltham, MA). From each normalized sample, 20 μl was combined into separate pools for fungal ITS fragments and 16S rRNA genes, concentrated using a SpeedVac, and resuspended in 90 μl of molecular grade water. From these suspensions, 45 μl was decanted, mixed with 15 μl loading dye, and run separately on 1.2% agarose gels (100 ml) precast with 3 μl of SYBR™ Safe DNA Gel Stain (Thermo Fisher Scientific, Waltham, MA). Target bands were cut out from the gel and cleaned using the PureLink™ Quick Gel Extraction Kit (Thermo Fisher Scientific, Waltham, MA), for a final pool volume of 30 μl. 16S rRNA gene and ITS pools were separately sequenced at the Cornell Genomics Facility (Ithaca, NY) on the Illumina MiSeq. A 500-cycle MiSeq Reagent Kit v.2 was used for both fungal ITS pool and prokaryotic 16S rRNA pool.

### Sequence Processing and Analysis

Initial sequence processing was conducted using DADA2 v.1.18 pipeline (Callahan et al., 2016). The taxonomic assignment was performed using the UNITE General FASTA release v.8.3 database (Kõljalg et al., 2005) for ITS gene sequences and Silva version 138.1 (Quast et al., 2012) database for prokaryotic 16S rRNA gene sequences.

All global analyses, including co-occurrence networks, linkage clustering, beta diversity, alpha diversity, and relative abundance were performed in R v. 3.5.3. All global analyses were conducted using rarefied read counts (based on the minimum available reads per sample among all samples). Rarefying helped in obtaining even numbers of reads by sample, thus normalizing inter-sample comparisons. In the case of ITS sequences, bulk soil, rhizosphere soil, and roots were rarefied to 11,113, 7,663, and 16,565 reads. With respect to 16S rRNA gene sequences, bulk soil, rhizosphere soil, and roots were rarefied to 3,754, 3,993, and 6,078 reads.

The co-occurrence networks containing both Fungal and prokaryotic taxa were constructed and analyzed using the SpiecEasi and Igraph packages in R (Csardi and Nepusz, 2006; Kurtz et al., 2015). Networks were constructed with ASVs that were present in 50% of samples or more. Network sparsity and stability were examined using SpiecEasi. The “spiec.easi” function was executed with the following specifications to construct all the networks: (i) method = “mb,” (ii) lambda.min.ratio = 1e^–2^, (iii) nlambda = 50, (iv) sel.criterion = “stars,” (v) pulsar.select = TRUE, (vi) rep.num = 99. As outlined in Agler et al. (2016), hub taxa were identified as those above the 90th percentile (1.3 standard deviations from the mean) of network ASVs for the measures of (i) betweenness centrality, (ii) hub scores (eigenvector centrality), and (iii) degree, for both Fungi and prokaryotes in that specific network. Following network construction in SpiecEasi and hub identification, networks were visualized with the ggnet2 function of the Ggally package of R (Schloerke et al., 2018). In order to test whether the layouts of the experimental networks were consistently and significantly different from stochastically created, scale-free networks with the same number of nodes as experimental networks, 100 stochastic networks were generated using Barbasi-Albert model of the “sample_pa” function in the igraph package of R. The degree distributions of random networks were also compared to those of experimental networks using the non-parametric two sample Kolmogorov-Smirnov using the “ks.test” function in the stats package of R. The “sample_pa” and “ks.test” functions have been previously used in a number of articles including (Longley et al., 2020).

The Bray-Curtis dissimilarities between samples were used to perform principal coordinates analyses (PCoA) to investigate β-diversity (OTU diversity between samples). Permutational multivariate analysis of variance (PERMANOVA) was performed using Bray-Curtis dissimilarity to identify the factors/explanatory variables (=Site type, soybean growth stage) that significantly contributed to total observed variation in PCoA plots. Significance was assessed from 999 permutations. PERMANOVA was performed separately for bulk soil, rhizosphere soil, and roots.

Alpha diversity was estimated for each sample using the Inverse Simpson index and Chao1 richness within the *vegan* R package. Subsequently, analysis of variance (ANOVA) was performed using generalized linear mixed model (GLMM) approach in SAS version 9.4 (SAS^®^ Institute, 2017) to test the main/simple effects (α = 0.05) of site type, soybean growth stage, and sample type (fixed factors) on two alpha diversity measures. Location was considered as a random factor. As both measures were count data, modeling was performed with the negative binomial distribution and using integral approximations to the likelihood. The following specifications were used for modeling: (1) link function = Log; (2) variance component estimation method = Maximum Likelihood; (3) degrees of freedom method = Residual; (4) non-linear parameter optimization method = Newton-Raphson with Ridging; (5) overdispersion fixation method = Laplace. The *inverse link* function was used to create means and associated standard errors at the data scale.

Relative abundance of taxonomic groups (phylum and genus level) was determined using the *transform_sample_counts ()* function in the R package *phyloseq*. Heatmaps and dendrograms based on average linkage clustering of ASV relative abundance were generated with the *heatmap()* function in R package *Heatplus*. For both fungal and prokaryote analyses, ASVs with >2% relative abundance were included in the analysis.

## Results

### Structure of Microbial Co-occurrence Networks

**FIGURE 2:**
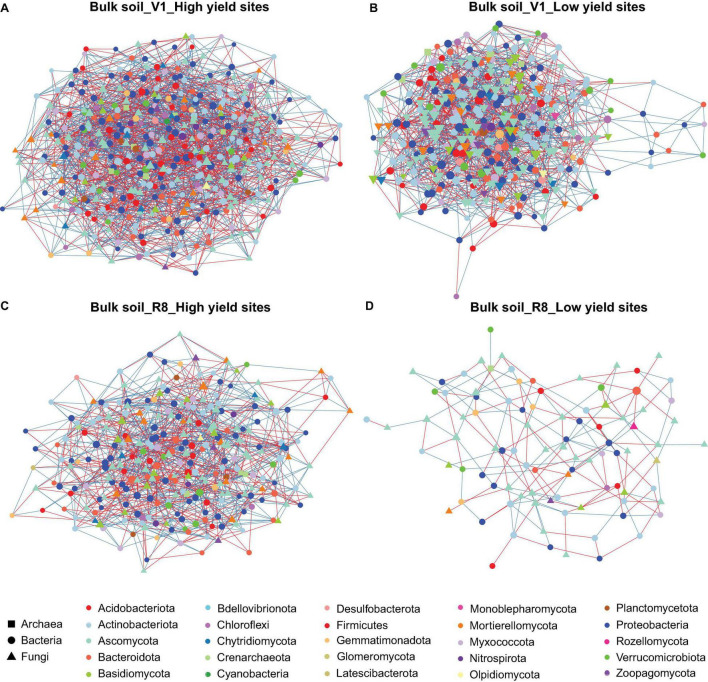
Network of taxon associations for bulk soil samples. Co-occurrence of fungal and prokaryotic taxa for high yield sites at V1 soybean growth stage **(A)**, low yield sites at V1 soybean growth stage **(B)**, high yield sites at R8 soybean growth stage **(C)**, and low yield sites at R8 soybean growth stage **(D)**. Nodes represent exact sequence variants (ESVs). Colors represent specific phyla. Shapes represent specific domains. Red solid lines (edges/links) connecting nodes indicate statistically significant negative correlations, and solid blue lines, positive correlations between the connected taxa. Node sizes are proportional to the degree (=node connectivity). Nodes with degree ≥5 were included in the network for the purpose of visualization clarity.

**FIGURE 3:**
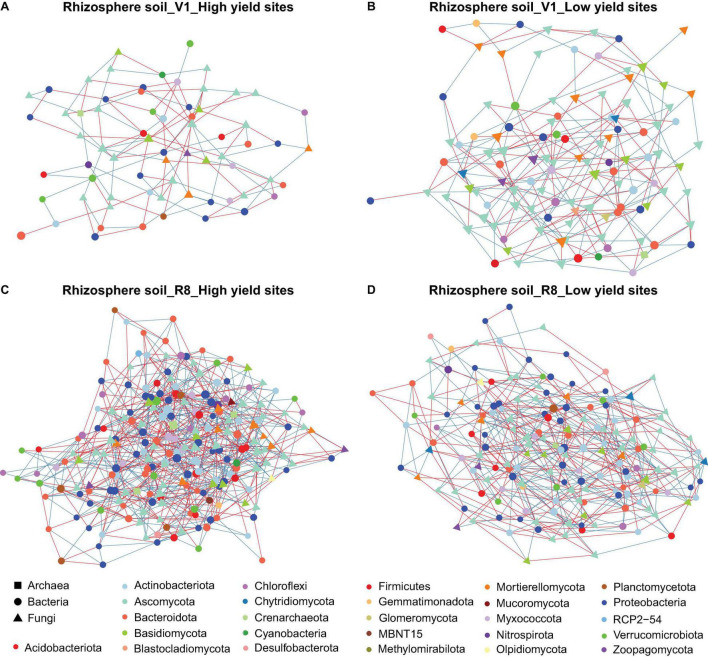
Network of taxon associations for rhizosphere soil samples. Co-occurrence of fungal and prokaryotic taxa for high yield sites at V1 soybean growth stage **(A)**, low yield sites at V1 soybean growth stage **(B)**, high yield sites at R8 soybean growth stage **(C)**, and low yield sites at R8 soybean growth stage **(D)**. Nodes represent exact sequence variants (ESVs). Colors represent specific phyla. Shapes represent specific domains. Red solid lines (edges/links) connecting nodes indicate statistically significant negative correlations, and solid blue lines, positive correlations between the connected taxa. Node sizes are proportional to the degree (=node connectivity). Nodes with degree ≥5 were included in the network for the purpose of visualization clarity.

**FIGURE 4:**
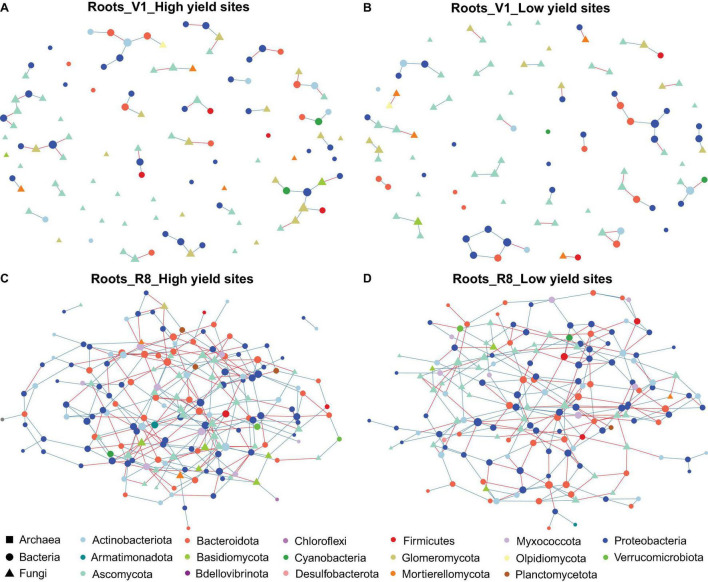
Network of taxon associations for root samples. Co-occurrence of fungal and prokaryotic taxa for high yield sites at V1 soybean growth stage **(A)**, low yield sites at V1 soybean growth stage **(B)**, high yield sites at R8 soybean growth stage **(C)**, and low yield sites at R8 soybean growth stage **(D)**. Nodes represent exact sequence variants (ESVs). Colors represent specific phyla. Shapes represent specific domains. Red solid lines (edges/links) connecting nodes indicate statistically significant negative correlations, and solid blue lines, positive correlations between the connected taxa. Node sizes are proportional to the degree (=node connectivity).

Microbial networks constructed for three sample types from high and low yield sites at two SGS differed in their network statistics (Table 1). Analyses performed with three sample types showed greater network size and total/positive/negative edges for high yield sites compared to low yield sites at both V1 and R8 growth stages, with the exception of rhizosphere soil at V1 stage (Table 1 and Figures 2A–D). All networks contained a greater number of prokaryotic than fungal nodes, except for root networks at V1 stage from both site types. Overall, networks had a diverse mix of bacterial and fungal phyla. In case of bacteria, bulk soil, rhizosphere soil, and root networks were dominated by Proteobacteria, Actinobacteriota, and Bacteroidota (Figures 2A–D, 3A–D, 4A–D). Fungal nodes were primarily from Ascomycota, Basidiomycota, and Mortierellomycota (Figures 2A–D, 3A–D, 4A–D) for bulk and rhizosphere soil networks. For root networks, Ascomycota and Glomeromycota were predominant in V1 networks while Ascomycota and Basidiomycota were predominant in R8 networks. When compared to 100 stocastic networks, each network except the rhizosphere soil-high sites-R8 and roots-low sites-V1 networks consistently had a significantly (*p* < 0.05) different network layouts than 100 stochastic networks (Table 1).

**TABLE 1:** Attributes describing the bipartite microbial networks for different sample types collected from high and low yield sites across eight Pennsylvania farms at V1 (one set of unfolded trifoliate leaf is visible) and R8 (95% of the pods have reached their mature color) soybean growth stages.

Network	# Nodes			# Edges			# Hubs			#Modules	Stability	Sparsity	*P*-value*	*P*-value range^±^
	Total	Fungal	Prokaryotic	Total	Positive	Negative	Total	Fungal	Prokaryotic					
Bulk soil: low sites at V1	374	122	252	1,644	950	694	15	7	8	26	0.0494	0.0235	0.0019	9.86E-06–0.0140
Bulk soil: high sites at V1	419	123	296	2,066	1,119	947	24	8	16	22	0.0496	0.0235	0.0237	5.35E-05–0.0470
Bulk soil: low sites at R8	212	81	131	464	272	192	11	6	5	20	0.0479	0.0206	0.0241	0.0042–0.0330
Bulk soil: high sites at R8	313	96	217	1,159	648	511	14	5	9	23	0.0496	0.0237	0.0193	7.85E-04–0.0428
Rhizo. soil: low sites at V1	204	90	114	464	273	191	5	3	2	31	0.0480	0.0223	0.0048	0.0027–0.0226
Rhizo. soil: high sites at V1	193	81	112	385	214	171	8	6	2	28	0.0490	0.0207	0.0116	0.0077–0.0461
Rhizo. soil: low sites at R8	241	79	162	678	366	312	13	6	7	24	0.0495	0.0233	0.0006	0.0136–0.0365
Rhizo. soil: high sites at R8	274	82	192	890	488	402	12	2	10	28	0.0493	0.0237	0.0049	0.0434–0.2397
Roots: low sites at V1	96	53	43	52	34	18	3	0	3	46	0.0464	0.0113	0.1066	0.0174–0.1809
Roots: high sites at V1	110	57	53	56	35	21	2	1	1	54	0.0440	0.0093	0.0351	0.0036–0.0333
Roots: low sites at R8	163	35	128	264	155	109	5	1	4	18	0.0488	0.0199	0.1148	0.0096–0.0389
Roots: high sites at R8	177	37	140	293	171	122	9	3	6	26	0.0470	0.0187	0.0046	0.0054–0.0438

*P-value indicates the probability value obtained from two sample Kolmogorov-Smirnov tests to assess the significance of the node degree distribution between random networks and the of experimental network of interest. ^±^P-value range represent the range of computed p-values when comparing the experimental network against 100 random networks. All random networks were created with the same number of nodes as experimental networks.

The co-occurrence analyses for bulk soils showed a reduction in network size and number of edges from V1 to R8 SGS for both site types (Table 1 and Figures 2A–D). However, for rhizosphere soil and roots from both site types, the network size as well as the number of edges increased from V1 to R8 (Figures 3A–D, 4A–D). Out of 118 hub ASVs detected in 12 networks (two site types, three sample types, and two SGS), 89 were restricted to a single network (Supplementary Table 1). Certain hub species were present in multiple networks. For example, the fungus *Corynespora cassiicola* was a hub in networks created for both site types with rhizosphere soil and roots from R8 soybean growth stage. Most prokaryotic hubs consisted of Proteobacteria and Actinobacteriota while fungal hubs were mainly comprised of Ascomycota and Basidiomycota (Supplementary Table 1). For bulk soil, high yield site network showed a greater number of hubs compared to that of low yield site network at both growth stages (Table 1). For, rhizosphere soil, although high yield site network showed a greater number of hubs compared to that of low yield site network at V1 growth stage, the opposite was observed at R8 growth stage. For, roots, high yield site network showed a lower number of hubs compared to that of low yield site network at V1 growth stage. However, the opposite was observed at R8 growth stage. *Bradyrhizobium elkanii*, which is the most common symbiotic nitrogen fixer associated with soybean in the continental United States, was present in networks created for all sample types (bulk/rhizosphere soil and roots). However, it was not detected as a hub in any of the networks (Supplementary Table 1).

### Beta-Diversity Analysis of Fungal and Prokaryotic Composition

**FIGURE 5:**
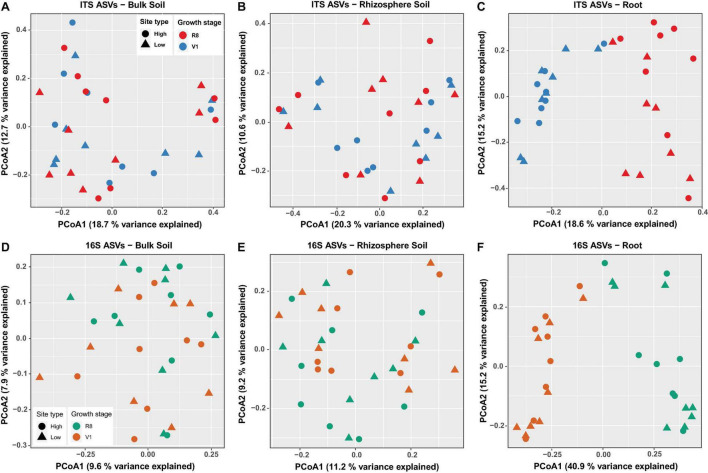
Principal Coordinates Analysis (PCoA) of ASVs based on Bray-Curtis dissimilarity for ITS-bulk soil **(A)**, ITS-rhizosphere soil **(B)**, ITS-roots **(C)**, 16S rRNA-bulk soil **(D)**, 16S rRNA-rhizosphere soil **(E)**, and 16S rRNA-roots **(F)**. Marker shape and color indicate site type (high/low yield) and soybean growth stage (V1 = one set of unfolded trifoliate leaf is visible; R8 = 95% of the pods have reached their mature color), respectively.

Principal coordinates analyses (PCoA) of fungal data using Bray-Curtis dissimilarity values showed that the percent variation explained by the first two principal coordinates was greatest for root samples (33.8%) followed by bulk soil (31.4%) and rhizosphere soil samples (30.9%) (Figures 5A–C). Neither bulk soil nor rhizosphere soil samples were appeared to cluster based on site type (high/low yield) or soybean growth stage (Figures 5A,B). However, a clear grouping was observed among root samples based on soybean growth stage but not based on site type (Figure 5C). PERMANOVA showed non-significant effect of site type on the observed total variation for all sample types (bulk soil: *R*^2^ = 0.03, *P* = 0.6753; rhizosphere soil: *R*^2^ = 0.02, *P* = 0.9540; roots: *R*^2^ = 0.02, *P* = 0.8621). The effect of soybean growth stage on the observed total variation was non-significant for bulk soil samples (*R*^2^ = 0.03, *P* = 0.7173) and rhizosphere soil samples (*R*^2^ = 0.04, *P* = 0.1459). However, a significant growth stage effect was evident for root samples (*R*^2^ = 0.16, *P* < 0.0001).

For prokaryotic data, the percent variation explained by the first two principal coordinates was similar for bulk and rhizosphere soil samples (17.5 and 20.4%, respectively), while it was greater for root samples (56.1%) (Figures 5D–F). Bulk and rhizosphere soil samples did not cluster based on site type or soybean growth stage (Figures 5D,E). A clear grouping was observed among root samples based only on soybean growth stage but not based on site type (Figure 5F). PERMANOVA revealed non-significant effect of site type on the observed total variation for all sample types (bulk soil: *R*^2^ = 0.03, *P* = 0.7952; rhizosphere soil: *R*^2^ = 0.03, *P* = 0.8591; roots: *R*^2^ = 0.02, *P* = 0.8741). The effect of soybean growth stage on the observed total variation was non-significant for bulk soil samples (*R*^2^ = 0.04, *P* = 0.0769). However, a significant growth stage effect was evident for rhizosphere soil (*R*^2^ = 0.06, *P* = 0.0020) and root samples (*R*^2^ = 0.36, *P* < 0.001).

### Heatmap Analysis With Average Linkage Clustering

**FIGURE 6:**
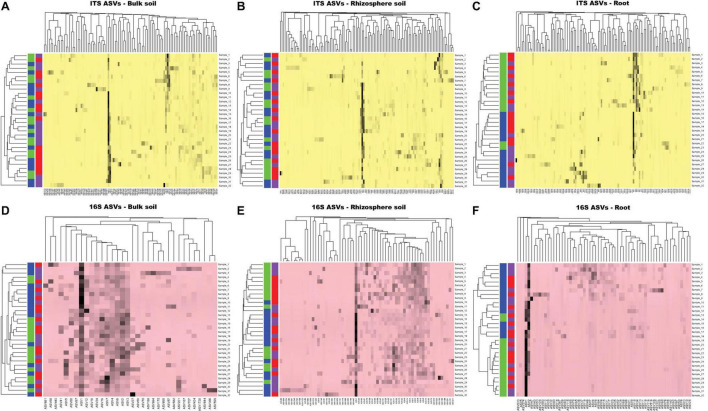
The heat map analysis of ASVs based on average linkage clustering for ITS-bulk soil **(A)**, ITS-rhizosphere soil **(B)**, ITS-roots **(C)**, 16S rRNA-bulk soil **(D)**, 16S rRNA-rhizosphere soil **(E)**, and 16S rRNA-roots **(F)**. The purple, red, green, and blue color boxes indicate the low yield site, high yield site, V1 soybean growth stage (one set of unfolded trifoliate leaf is visible), and R8 soybean growth stage (95% of the pods have reached their mature color), respectively. Note that, based on both ITS and 16S rRNA OTU data sets, there is no clear clustering of samples (bulk soil, rhizosphere soil, and soybean roots) based on either site type or soybean growth stage.

Based on ASVs >2% abundance criterion, 103, 115, and 115 fungal ASVs were included in the heatmap analysis for bulk soil, rhizosphere soil, and roots, respectively. Similarly, 34, 65, and 63 prokaryotic ASVs were included in the analysis for bulk soil, rhizosphere soil, and roots, respectively. Analysis with fungal as well as prokaryotic ASVs did not cluster bulk soil samples into two distinct groups based on either site type or soybean growth stage (V1 and R8) (Figures 6A–F). Similar results were observed for rhizosphere soil samples. Although not distinctly grouped into two groups, root samples showed some degree of clustering based on soybean growth stage with both fungal and prokaryotic ASV data (Figures 6C,F).

### Alpha-Diversity Analysis of Fungal and Prokaryotic Composition

**FIGURE 7:**
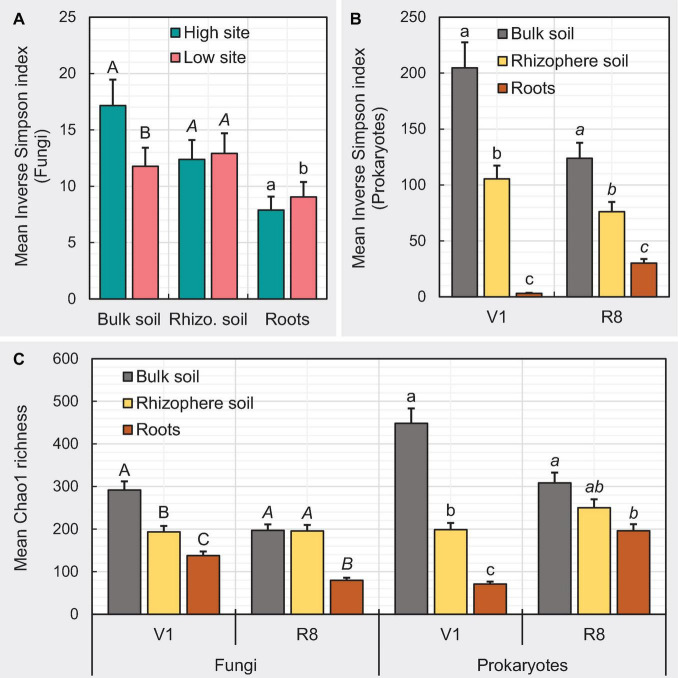
Bar graphs depicting the impact of studied variables on mean α-diversity measures. Designation of the significant mean difference in panel **(A)** was based on Fisher’s least significant difference (LSD). Same for panels **(B,C)** were based on the adjustment for multiple comparisons using Tukey-Kramer test at the 5% level of significance (=5% experiment wise error rate). Error bars represent standard errors. Means followed by a common letter within each letter type (i.e., uppercase, lowercase, and italic) are not significantly different. V1 = one set of unfolded trifoliate leaf is visible; R8 = 95% of the pods have reached their mature color.

Here we measured diversity within samples (i.e., how different are ASVs within samples) using Inverse Simpson index, which accounts for both the number of ASVs observed and evenness in the relative abundance of ASVs, where higher index values imply greater diversity and vice versa. We also used Chao1 index, which is a measure of the number of different ASVs present in a given sample. For fungal ASVs, ANOVA showed a significant main effect of soybean growth stage (*P* = 0.0016) and sample type × site type interaction effect (*P* = 0.0412) on mean Inverse Simpson index. The mean Inverse Simpson index for fungal ASVs was significantly greater (*P* = 0.0016) at V1 growth stage (9.94 ± 1.12) compared to that of at R8 stage (13.27 ± 1.45). Although the mean Inverse Simpson index for rhizosphere soil and roots from two site types did not significantly differ, bulk soil from high yield sites showed a significantly greater index than that from the low yield sites (Figure 7A). Per the ANOVA, sample type × growth stage interaction effect was significant (*P* < 0.0001) on the mean Chao1 richness. At V1 growth stage, Chao1 richness was greater in bulk soil compared to rhizosphere soil (*P* < 0.0001) and roots (*P* < 0.0001) while the Chao1 richness of rhizosphere soil was significantly greater than roots (*P* < 0.0001) (Figure 7C). At R8 growth stage, the Chao1 richness of bulk soil (*P* < 0.0001) and rhizosphere soil (*P* < 0.0001) was greater compared to that of roots (Figure 7C).

For prokaryotic ASVs, the sample type × growth stage interaction effect was significant (*P* < 0.0001) on Inverse Simpson index. At both V1 and R8 growth stages, the Inverse Simpson index was greater in bulk soil compared to rhizosphere soil (*P* < 0.0001) and roots (*P* < 0.0001) while the same of rhizosphere soil was significantly greater than roots (*P* < 0.0001) (Figure 7B). The sample type × growth stage interaction effect was also significant (*P* < 0.0001) on the mean Chao1 richness. At V1 growth stage, Chao1 richness was greater in bulk soil compared to rhizosphere soil (*P* < 0.0001) and roots (*P* < 0.0001) while the same of rhizosphere soil was significantly greater than roots (*P* < 0.0001) (Figure 7C). At R8 growth stage, the Chao1 richness of bulk soil (*P* = 0.0002) was greater than that of roots (Figure 7C).

### Relative Abundance of Fungal and Prokaryotic Taxa

**FIGURE 8:**
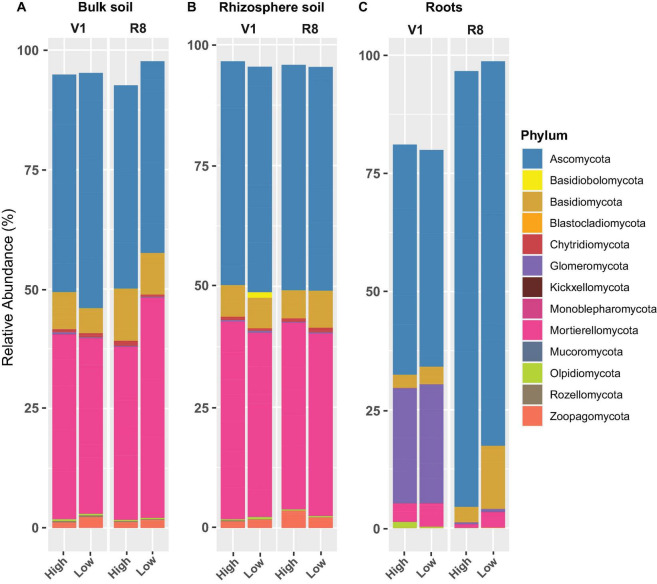
Stacked bar plots showing the mean relative abundance of major fungal phyla for **(A)** bulk soil, **(B)** rhizosphere soil, and **(C)** soybean root samples collected from high and low yield sites at V1 (one set of unfolded trifoliate leaf is visible) and R8 (95% of the pods have reached their mature color) soybean growth stages across eight soybean farms in Pennsylvania.

Phylum-level fungal abundance analysis showed that Ascomycota was the predominant phylum in all sample types at both growth stages and site types (Figures 8A–C). For bulk and rhizosphere soils, the relative abundance of Ascomycetes appeared to be relatively similar at both SGS for both site types; nonetheless, it tended to increase in roots from V1 to R8 at both site types. Further, the Ascomycota abundance in roots from high yield sites was greater than that of low yield sites at both growth stages. On the contrary, the abundance of Mortierellomycota and Basidiomycota in roots from high yield sites was lower than that of low yield sites at both growth stages. Although Zoopagomycota was prominent in both soil types, it was less prominent in roots. Glomeromycota was very prominent in roots, particularly at V1 growth stage.

**FIGURE 9:**
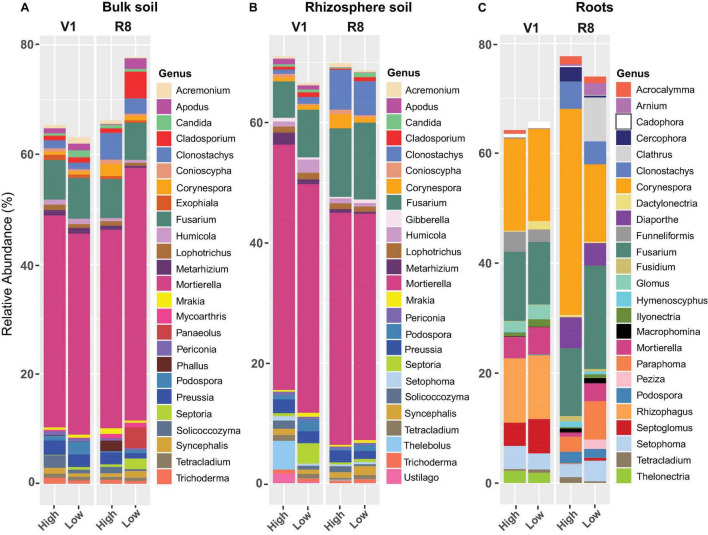
Stacked bar plots showing the mean relative abundance of major fungal genera for **(A)** bulk soil, **(B)** rhizosphere soil, and **(C)** soybean root samples collected from high and low yield sites at V1 (one set of unfolded trifoliate leaf is visible) and R8 (95% of the pods have reached their mature color) soybean growth stages across eight soybean farms in Pennsylvania.

Genus level fungal abundance analysis showed that the relative abundance of *Apodus*, *Cladosporium*, *Fusarium*, *Podospora*, and *Septoria*, was greater in bulk soil from low yield sites compared to high yield sites at both SGS (Figure 9A). Abundance of *Clonostachys*, *Corynespora*, *Exophiala*, *Humicola*, *Lophotrichus*, *Metarhizium*, *Solicoccozyma*, and *Trichoderma* was greater in bulk soil from high yield sites compared to low yield sites at both growth stages.

For rhizosphere soil, low yield sites contained greater abundance of *Cladosporium*, *Fusarium*, *Mrakia*, *Podospora*, and *Septoria* than high yield sites at both growth stages (Figure 9B). The abundance of *Conioscypha*, *Corynespora*, *Metarhizium, Preussia*, and *Solicoccozyma* was greater in high yield sites than low yield sites at both growth stages.

Soybean roots from low yield sites had greater abundance of *Ilyonectria*, *Macrophomina*, *Mortierella*, and *Septoglomus* compared to those in high yield sites at both growth stages while the abundance of *Fusarium* and *Rhizophagus* was greater at R8 (Figure 9C). *Acrocalymma*, *Corynespora*, and *Podospora* were more abundant in roots from high yield sites than low yield sites at both growth stages, while *Cercophora*, *Clonostachys*, *Diaporthe*, and *Hymenoscyphus* were present in greater abundance at R8. For high yield sites, abundance of root associated *Corynespora* increased from V1 to R8, but it decreased in roots from low yield sites.

**FIGURE 10:**
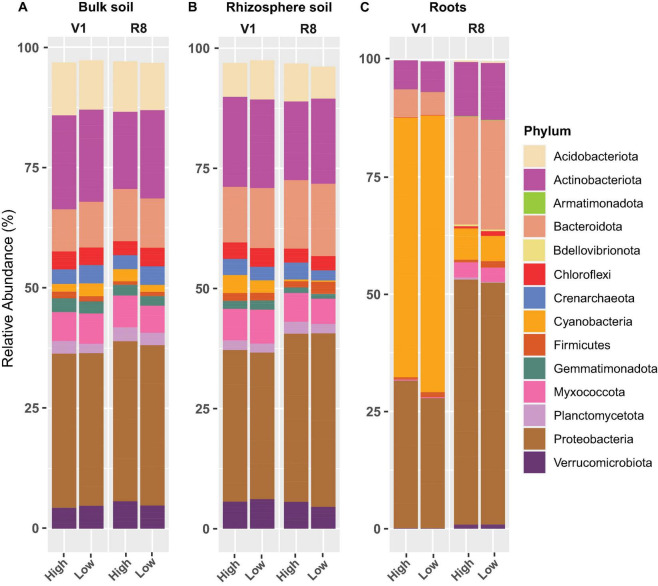
Stacked bar plots showing the mean relative abundance of major prokaryotic phyla for **(A)** bulk soil, **(B)** rhizosphere soil, and **(C)** soybean root samples collected from high and low yield sites at V1 (one set of unfolded trifoliate leaf is visible) and R8 (95% of the pods have reached their mature color) soybean growth stages across eight soybean farms in Pennsylvania.

Phylum-level prokaryotic abundance analysis showed that Proteobacteria was the predominant phylum in all sample types at both growth stages and site types (Figures 10A–C). Actinobacteriota and Bacteroidota were also abundant in all sample types. Although Acidobacteriota, Chloroflexi, Crenarchaeota, Gemmatimonadota, Planctomycetota, and Verrucomicrobiota were abundant in both soil types (across growth stages and site types), they were less conspicuous in roots. Cyanobacteria was prominent in roots, particularly at V1 stage.

**FIGURE 11:**
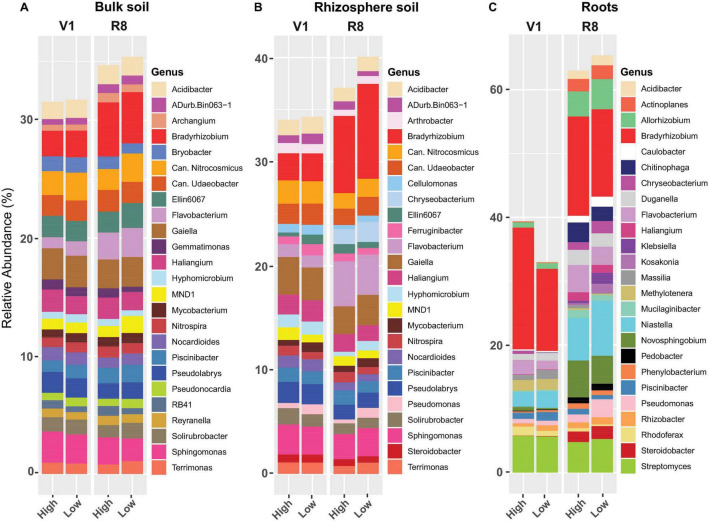
Stacked bar plots showing the mean relative abundance of major prokaryotic genera for **(A)** bulk soil, **(B)** rhizosphere soil, and **(C)** soybean root samples collected from high and low yield sites at V1 (one set of unfolded trifoliate leaf is visible) and R8 (95% of the pods have reached their mature color) soybean growth stages across eight soybean farms in Pennsylvania.

Genus-level prokaryotic abundance analysis showed that the relative abundance of major prokaryotic genera in both bulk and rhizosphere soils was similar between high and low yield sites at both growth stages (Figures 11A,B). Further, for majority of the genera, there was no marked difference between two site types at both growth stages. For roots, abundance of *Niastella*, *Piscinibacter*, *Pseudomonas*, and *Rhizobacter* from low yield sites was greater compared to high yield sites at both growth stages (Figure 11C). Abundance of *Bradyrhizobium*, *Flavobacterium*, *Novosphingobium*, and *Rhodoferax* was greater in roots from high yield sites compared to low yield sites at both SGS.

## Discussion

Microbial composition in soil and roots can influence plant physiological performance and yield. However, little is known about the association between the spatial variation of microbial composition and the corresponding spatial variation of crop yields at the site scale. Since many soil- and root-inhabiting fungal and prokaryotic taxa are uncultivable using conventional methods, a metabarcoding approach offers insights into populations only known by sequencing (Prosser, 2015). Our results, among the first using this approach to address such questions, showed that the abundance of key taxa and network structures of fungi and bacteria are different between high and low yield sites.

Network analysis is a powerful tool for investigating and recognizing patterns in large, complex datasets, which can be more challenging to detect using common methods in ecology like standard alpha/beta diversity analyses (Proulx et al., 2005). Our results showed greater network sizes and total/positive/negative edges for high yield sites compared to low yield sites at both growth stages for all sample types, except for the V1-rhizosphere soil network. These results indicated that microbial networks from high yield sites are more complex compared to those of low yield sites. A previous study by Zheng et al. (2021) showed that the healthy tobacco plants had more complex microbial networks than the plants with bacterial wilt caused by *Ralstonia solanacearum*. Although we did not visually observe any disease conditions in soybean plant from low yield sites, it is possible that less complex microbial networks contributed to less healthy plants that will eventually translate into poorer yields. More in-depth investigations are required to gain insights into mechanistic understanding on how greater network complexity and soybean yields.

*Bradyrhizobium elkanii* is reported as the major soybean nodulating rhizobia (Jordan, 1982). Although *B. elkanii* was present in all microbial co-occurrence networks constructed in the present study, it was not detected as a hub in any of the networks. This finding indicated that the potential interactions of *B. elkanii* with other prokaryotic/fungal members of the community is minimal, despite its importance in soybean cropping system. Poudel et al. (2016) discussed that the most important taxa for plant health may neither have any links to other taxa, nor have correlations with plant or pathogen performance components. Therefore, it was not surprising to see *B. elkanii* as a non-hub taxon in the current study.

Although the network association structure and related attributes tended to change depending on soybean growth stage, such changes did not appear to depend on site type. For instance, the network size and total/positive/negative edges increased from V1 to R8 for rhizosphere soil and roots for both high and low yield sites. Therefore, while there were temporally defined ecological rearrangements of microbial composition, as evidenced by the changes in topological attributes with the co-occurrence network, such changes appeared to be common between the two site types. As such, at least considering the seasonal scale, the temporal dynamics of microbial composition may not contribute to site-dependent spatial yield variation.

For both fungal and prokaryotic data, regardless of growth stage and site type, bulk soil showed the greatest Inverse Simpson index and Chao1 richness (measure of α-diversity), followed by rhizosphere soil and roots. As such, there was a diversity gradient from bulk soil to roots via the rhizosphere interface. Results were consistent with studies that have demonstrated the highest alpha diversity of both fungi and bacteria in the soil (Lebreton et al., 2019; Suárez-Moo et al., 2019).

As revealed by relative abundance analysis, fungal and prokaryotic composition varied based on site type (high/low), soybean growth stage (V1/R8), and sample type (bulk/rhizosphere soil, roots) at both phylum and genus level. Previous reports clearly demonstrated growth stage (Mougel et al., 2006; Houlden et al., 2008; Xu et al., 2009; Chaparro et al., 2014) and sample type (Sugiyama et al., 2014; Yurgel et al., 2017) dependent relative abundance variation of prokaryotic and fungal taxa.

Our findings showed a greater relative abundance of the fungal genus *Fusarium* in roots from low yield sites compared to high yield sites. Other than a single ASV that corresponds to an unclassified species of genus *Fusarium*, we detected *F. acutatum*, *F. solani*, *F. cuneirostrum*, and *F. oxysporum* in these root samples. Interestingly, all of these *Fusarium* species are known soybean pathogens (Daamen et al., 1991; Aoki et al., 2005; Zhang et al., 2010; Arias et al., 2013; Yan and Nelson, 2020; Degani and Kalman, 2021). We also found a greater relative abundance of the fungal species, *Macrophomina phaseolina* in roots from low yield sites compared to high yield sites. *M. phaseolina* is one of the most devastating pathogens in soybean causing charcoal rot disease (Mengistu et al., 2013; Bai et al., 2015; Bandara et al., 2020a). As such, soybean roots from low yield sites appeared to be under higher pathogen pressure and corresponding disease risks, although external disease symptoms such as rots, spots, and lesions were not visually observed during the sampling process.

Interestingly, the relative abundance of root-colonizing genus *Corynespora* (represented by a single species, *C. cassiicola*) was greater in high yield sites compared to low yield sites. This organism causes target spot on leaves, stems, roots and flowers of more than 280 plant species, including many economically important crops, such as soybean (Silva et al., 1995). However, Dixon et al. (2009) and Shimomoto et al. (2011) demonstrated greater pathogenicity variability among *C. cassiicola* isolates. It is possible that the pathogenicity of *C. cassiicola* from high yield sites is significantly lower, despite higher relative abundance. Alternatively, as an endophyte, *C. cassiicola* is reported to secrete an array of polyketide and fatty acid derivatives where their biological activities have not been tested (Chagas et al., 2015). These compounds can potentially be antifungal or antibacterial in nature. Given this possibility, greater relative abundance of *C. cassiicola* in roots from high yield sites could also be beneficial and contribute to greater yields. More investigations are essential to precisely determine the association between root colonizing *C. cassiicola* and soybean yields.

The bacterial genus, *Pseudomonas* was present in greater percentages, particularly in rhizosphere and root samples collected from low yield sites at both growth stages. Pseudomonads from rhizosphere soil was predominantly represented by *P. umsongensis* while *P. chlororaphis* and *P. frederiksbergensis* were the two species detected in roots. Most of the Pseudomonas species are pathogenic to plants (Young, 2010; Mansfield et al., 2012; Lamichhane et al., 2015). However certain *Pseudomonas* species such as *P. fluorescens* and *P. protegens* are useful as biocontrol agents (Ramette et al., 2011; Gull and Hafeez, 2012; Santoyo et al., 2012; Sivasakthi et al., 2014). Interestingly, the two species that we observed in greater quantities in roots from low yield sites are reported as antifungal and plant growth promoting (Bardas et al., 2009; Chatterjee et al., 2017). Although it seems counterintuitive to observe a greater number of beneficial Pseudomonads in roots from low yield sites compared to that of high yield sites, more in-depth studies could justifiably explain this phenomenon.

A noteworthy observation was the greater relative abundance of the genus *Flavobacterium* in roots from high yield sites compared to low yield sites at both SGS. Some *Flavobacterium* species are known as plant growth promoters (Sessitsch et al., 2004) while other species are reported to protect plants against microbial infections through root colonization (Sang and Kim, 2012) or bioremediation (Kang et al., 2013). Given these beneficial effects, greater *Flavobacterium* colonization in roots from high yield sites can in turn contribute to better physiological performance and greater yields.

The bulk soils from high yield sites at both SGS contained greater percentages of *Trichoderma* (predominantly *T. spirale* and an unclassified Trichoderma species) fungal genera. The beneficial mycoparasitic and other roles of various *Trichoderma* species are widely reported in relation to soybean production (Menendez and Godeas, 1998; Shovan et al., 2008; Bagwan, 2010; John et al., 2010; Khalili et al., 2016; Khaledi and Taheri, 2016; Zhang et al., 2016). Certain *Trichoderma* species can also control phytopathogenic nematodes like soybean root knot nematodes (Oyekanmi et al., 2007). In particular, the utility of *T. spirale* as a biocontrol agent against *C. cassiicola* is previously reported (Baiyee et al., 2019). Note that, as discussed above, the abundance of *C. cassiicola* was greater in high yield sites. Even if we assume that *C. cassiicola* isolates from high yield sites are pathogenic, the greater abundance of *T. spirale* in high yield sites can potentially suppress *C. cassiicola* and mitigate its negative impacts on soybean yields. Moreover, rhizosphere soils from high yield sites at both SGS contained greater percentages of genus *Metarhizium* (*M. anisopliae* and *M. marquandii*) compared to low yield sites. The beneficial entomopathogenic behavior of *Metarhizium* including *M. anisopliae* and *M. marquandii* is widely documented in relation to soybean production (Clifton et al., 2018; Baron et al., 2020; Lopes et al., 2020). Therefore, the greater occurrence of beneficial fungal genera such as *Trichoderma* and *Metarhizium* can potentially eases the soilborne pathogen and insect pressure. This could eventually translate into greater yields in high yield sites.

While the relative abundance of symbiotic nitrogen fixers like *Bradyrhizobium elkanii* was greater in roots from high yield sites at both V1 and R8 growth stages, free-living nitrogen fixers such as *Klebsiella* and *Kosakonia* were more abundant in roots from low yield sites compared to high yield sites. Symbiotic nitrogen fixation occurs under a narrow range of environmental conditions while free-living nitrogen fixation can occur under a wide range of environmental conditions (Smercina et al., 2019). In contrast to symbiotic fixation, free-living nitrogen fixation can take place even under non-optimal conditions such as greater oxygen concentration and lower carbon (energy) availability (Smercina et al., 2019). The greater abundance of free-living nitrogen fixers in low yield sites is therefore indicative of a less efficient nitrogen fixation via symbiotic fixers. Nevertheless, these symbiotic nitrogen fixers directly depend on the host (soybean plant in this case) to fulfill their carbon requirements. Given these observations, we hypothesize that soybean plants from low yield sites lose more carbon due to symbiotic nitrogen fixers while not getting enough fixed nitrogen, which in turn contributes to the poor yields. Furthermore, natural environments are ubiquitously inhabited by unproductive rhizobia strains that extort benefits without compensating costs and thus proliferate more efficiently than nitrogen-fixing cooperators (Gibson et al., 1975; Bottomley and Jenkins, 1983; Moawad et al., 1998; Burdon et al., 1999; Fujita et al., 2014). In the current study, the genus *Allorhizobium* (comprised of *A. cellulosilyticum*, *A. daejeonense*, *A. mesosinicum*, *A. phaseoli*, and two unclassified *Allorhizobium* species) was present in greater abundance in roots from low yield sites compared to that of high yield sites. The reports that reveal their use as efficient Nitrogen fixers are sparse. Therefore, we further hypothesize that different *Allorhizobium* species detected in the current study could be parasitic. The greater presence of potentially parasitic Allorhizobia in roots from low yield sites could use surplus energy for their own growth or for creating storage substances and in turn results in lower plant productivity and yields. A comprehensive comparison of Allorhizobia strains isolated from high and low yield sites in terms of their ability to affect soybean yields could provide additional insights into the relationship between site scale spatial variation of soybean yields and symbiotic nitrogen fixers.

## Conclusion

Spatial variation of crop yields exists even within small geographic regions (i.e., within a farm) is a commonly observed phenomenon. Several factors can contribute to such variation including soil microbiota. Recent advances in high-throughput sequencing have allowed us to explore microbial composition in much greater detail, as we can identify even poorly known phyla with limited phenotypic data. Using an amplicon sequencing approach, we found that high yield sites contain significantly fewer fungal genera that contain known pathogenic species and more fungal genera that contain species that are known to be mycoparasitic or nematophagous. Moreover, the number of root-colonizing bacterial genera that contain known plant growth promoting bacteria was also greater in high yield sites. The greater occurrence of free-living nitrogen fixing bacterial genera in roots from low yield sites indicates that prevailing edaphic conditions in low yield sites were not ideal for symbiotic nitrogen fixation. Additionally, results from microbial network analysis showed that the size and the total number of edges in networks from high yield sites is greater with a larger number of specific biological interactions among them. Therefore, our findings showed that the occurrence of high and low yield spots in soybean fields was associated with a consortium of microbial taxa suggesting the importance of certain microbial taxa to the establishment and stability of healthy soil. Taken together, our findings provide new insights into the relationship between fungal and prokaryotic composition to observed site-scale spatial heterogeneity in soybean yield. These findings will be helpful in devising future site-specific management practices of soil and root-associated microorganisms toward better soil health and increased agricultural production.

## Data Availability Statement

The datasets generated for this study can be found in the NCBI SRA Archive: PRJNA707296.

## Author Contributions

PE and TB secured the funding and edited the manuscript. PE conceptualized and supervised the project. AB and PE designed the experiments. PE and DW coordinated the sample collection. AB and DW processed the samples. AB, DW, and RT prepared the amplicon libraries for sequencing. AB analyzed the data, created the figures, and wrote the draft manuscript. All authors approved the final manuscript.

## Conflict of Interest

The authors declare that the research was conducted in the absence of any commercial or financial relationships that could be construed as a potential conflict of interest.

## Publisher’s Note

All claims expressed in this article are solely those of the authors and do not necessarily represent those of their affiliated organizations, or those of the publisher, the editors and the reviewers. Any product that may be evaluated in this article, or claim that may be made by its manufacturer, is not guaranteed or endorsed by the publisher.
